# Supplementary material for: A modelling study of hole transport in GaN/AlGaN superlattices
Source: Sci Rep. 2023 Nov 16;13:20053. doi: 10.1038/s41598-023-47345-9 (PMC10654525; doi:10.1038/s41598-023-47345-9)
Supplement: Supplementary file 1 — Supplementary Information. [file 41598_2023_47345_MOESM1_ESM.pdf]

Table: The parameters for bulk material in simulation

	GaN	AlN
Energy gap(eV)[27]	3.4	6.2
State dielectric constant(ε_0)[23]	10	8.5
Effective heavy hole mass(xy)[27]	$1.6\mathrm{m}_0$	$10.42\mathrm{m}_0$
Effective heavy hole mass(z)[27]	$1.1\mathrm{m}_0$	$3.53\mathrm{m}_0$
Effective light hole mass(xy)[27]	$0.15\mathrm{m}_0$	$0.24\mathrm{m}_0$
Effective light hole mass(z)[27]	$1.1\mathrm{m}_0$	$3.53\mathrm{m}_0$
Effective mass of density of state[27]	$1.5\mathrm{m}_0$	$7.26\mathrm{m}_0$
Strain [23]	0 (Based on sapphire)	2.48%
Band offset(eV)[27]	0	0.85
Lattice constant(nm)[26]	0.52	0.5
Acceptor ionization energy(meV)[25]	170	517
Piezoelectric constant($10^{-12}\mathrm{m/V}$[23]	d31=-1.7<br>d15=-1.7<br>d33=3.4	d31=-2<br>d15=-2<br>d33=4
Spontaneous polarization constant($\mathrm{C/m^2}$)[23]	-0.029	-0.081

Legend:

- The values provided in the table are based on the references indicated (e.g., [27], [23]).
- "m_0" denotes the free electron rest mass.
- "xy" and "z" denotes the lattice direction
- The piezoelectric constants are given in three different orientations: d31, d15, and d33.
- The strain value for GaN is based on its growth on a sapphire substrate.
